# Comparative PIV and LDA studies of Newtonian and non-Newtonian flows in an agitated tank

**DOI:** 10.1007/s11696-017-0307-4

**Published:** 2017-10-04

**Authors:** Anna Story, Zdzisław Jaworski, Mark J. Simmons, Emilia Nowak

**Affiliations:** 1 Institute of Chemical Engineering and Environmental Protection Processes, West Pomeranian University of Technology, Aleja Piastów 42, 71-065 Szczecin, Poland; 2 School of Chemical Engineering, The University of Birmingham, Edgbaston, Birmingham, B15 2TT UK; 3 School of Food and Nutrition, Massey University, Albany, Auckland 0745 New Zealand

**Keywords:** Particle image velocimetry, Viscoelastic fluid, Stirred tank, PMT impeller, Laser Doppler anemometry

## Abstract

The paper presents results of an experimental study of the fluid velocity field in a stirred tank equipped with a Prochem Maxflo T (PMT) type impeller which was rotating at a constant frequency of *N* = 4.1 or 8.2 s^−1^ inducing transitional (*Re* = 499 or 1307) or turbulent (*Re* = 2.43 × 10^4^) flow of the fluid. The experiments were performed for a Newtonian fluid (water) and a non-Newtonian fluid (0.2 wt% aqueous solution of carboxymethyl cellulose, CMC) exhibiting mild viscoelastic properties. Measurements were carried out using laser light scattering on tracer particles which follow the flow (2-D PIV). For both the water and the CMC solution one primary and two secondary circulation loops were observed within the fluid volume; however, the secondary loops were characterized by much lower intensity. The applied PMT-type impeller produced in the Newtonian fluid an axial primary flow, whilst in the non-Newtonian fluid the flow was more radial. The results obtained in the form of the local mean velocity components were in satisfactory agreement with the literature data from LDA. Distribution of the shear rate in the studied system was also analyzed. For the non-Newtonian fluid an area was computed where the elastic force dominates over the viscous one. The area was nearly matching the region occupied by the primary circulation loop.

## Introduction

The selection of the correct type of impeller for implementation in a mixing process is very important to ensure process requirements and objectives are met. Therefore, basic knowledge about the flow field is needed in order to optimize the mixing tank design as well as assessment of its practical applications. It should be noted that the flow field generated in a mixing tank depends not only on the impeller type and shape of the tank, but also on the rheological parameters of the stirred medium. A large number of published papers presenting results of the study of the flow field in stirred tanks are focused on the flow of Newtonian fluids (Kysela et al. 2014a, b; Li et al. 2011). However, in industrial practice, non-Newtonian fluids are most commonly used, including polymer solutions and alloys, plastics, paints, varnishes, bitumen, oil, tar, blood and many other fluids (Ferguson and Kembłowski 1991), which are rheologically more complex than the Newtonian fluids. The most common deviation of the rheology of the non-Newtonian fluid from the Newtonian fluid behavior is that the viscosity and elasticity depend upon the shear rate.

Many research works are solved assuming completely viscous behavior of the liquid. However, sheared viscoelastic fluids exhibit not only shear stresses, *τ*, but also normal stresses, *σ*, which act in the direction perpendicular to the fluid flow direction. A common phenomenon caused by the normal stresses is, for example, rod climbing during the mixing operation; this is called the Weissenberg effect (Weissenberg and Freeman 1946). Thus, the liquid can move against gravity and centrifugal force. Components of the normal stress tensor are not equal, resulting in the introduction of a preferential flow orientation and consequently its anisotropy (Ferguson and Kembłowski 1991). Elasticity of the liquid is quantitatively characterized by the Weissenberg criterion, We, also known as a recoverable shear strain (Ferguson and Kembłowski 1991) connecting two types of stresses:

1 $$We = \frac{{N_{1} }}{2\tau },$$

where *N*_1_ is the first normal stress difference and *τ* is the shear stress. Literature reports that a fluid is considered to be highly elastic when the Weissenberg criterion exceeds 0.5 (Ferguson and Kembłowski 1991) and then the *N*_1_ > *τ*. Research related to the viscoelastic fluid flow was performed by many researchers (Seyssiecq et al. 2003; Jahangiri 2005, 2006, 2007, 2008; Anne-Archard and Boisson 1995; Youcefi et al. 1997; Ramsay et al. 2016), but only a few works focused on results related to the flow field generated during mechanical stirring of that group of non-Newtonian fluids. For example, Jahangiri in his publications (Jahangiri 2005, 2006, 2007, 2008) presented results of an experimental study using a laser Doppler anemometry (LDA) technique for the transient flow of a viscoelastic fluid. The mixing tank was equipped with a high-speed Rushton turbine (Jahangiri 2005, 2006) or a low-speed helical ribbon impeller (Jahangiri 2007, 2008).

Elastic properties of viscoelastic fluids increase with increasing shear rate and therefore they will be more pronounced in those regions of the mixed fluid where also high shear forces exist. Such regions appear near the impeller region and tank wall, where high velocity gradients also occur. This issue can be important for the wall heat transfer in stirred tanks. Elasticity undoubtedly affects the flow field generated during mechanical mixing of a viscoelastic fluid; therefore, the main aim of this study was to obtain experimental information about the flow field.

This study was performed in a stirred tank filled with either Newtonian or mildly viscoelastic fluid, equipped with a six-blade Prochem Maxflo T (PMT) type impeller. The PMT impeller was often applied in experimental studies of bioprocesses (Schell et al. 2001; McFarlane et al. 1995; McFarlane and Nienow 1996a, b). Other authors (Jaworski et al. 1996; Jaworski and Nienow 1994) applied LDA measurements in analysis of the velocity field generated by the PMT impeller in a stirred tank filled with liquids of different rheological properties. Particle image velocimetry (PIV) was used in none of the analyzed publications presenting results in a system with the PMT impeller; therefore, the aim of this work is to fill this gap in the literature.

Results of the intended PIV measurements, in terms of mean fluid velocity, can be compared with literature results from LDA (Jaworski and Nienow 1994). A similar comparison of results related to mechanical mixing and obtained from these two experimental techniques was performed by other authors (Kysela et al. 2014a, b; Li et al. 2011); however, in all cases the system was equipped with Rushton turbine and the tested liquid was water. In this study, experiments should be carried out for both Newtonian and non-Newtonian fluids and the obtained results should be analyzed to give information how the rheological properties of the stirred medium and the impeller speed affect the mean fluid velocity and also the shear rate.

The collected data constitute a basis for evaluating the practical application of the PMT-type impeller in real systems encountered in industry, where processes with highly shear-thinning and viscoelastic fluids are commonplace. They also illustrate how the non-Newtonian flow properties play an important role, which implies that they must be properly taken into account while predicting the flow field in numerical simulation.

## Theoretical

The fluid flow in the mechanical mixers is three-dimensional and is characterized by continuous fluctuations of all the velocity components, both in space and in time. If the fluid flow is turbulent or otherwise unstable, but is steady on average, according to the Reynolds hypothesis it can be regarded as the superposition of a mean flow with a fluctuating component. Therefore, the instantaneous fluid velocity, $v_{i}$, can be decomposed as the sum of the mean, $\bar{v}_{i}$, and fluctuating, $v'_{i}$, velocity (Pope 2000):

2 $$v_{i} = \bar{v}_{i} + v'_{i} \quad {\text{with}}\;i = r,\theta ,z,$$

where the mean velocity, $\bar{v}_{i}$, is calculated on the basis of a set of *M* experimental values of the instantaneous velocity, $v_{i,m}$:

3 $$\bar{v}_{i} = \frac{1}{M}\mathop \sum \limits_{m = 1}^{M} v_{i,m} .$$

The fluctuating velocity is defined as deviation of the instantaneous velocity from the mean velocity. In practice, the average value of the fluctuating velocity components is calculated as a standard deviation, i.e., the square root of the variance:

4 $$v'_{i} = \sqrt {\sigma^{2} } = \sqrt {\frac{{\mathop \sum \nolimits_{m = 1}^{M} \left( {v_{i,m} - \bar{v}_{i} } \right)^{2} }}{M - 1}} .$$

In the case of laminar fluid flow the fluctuating velocity component is equal to zero and Eq. (2) is redundant; however, the majority of mechanical mixing processes in stirred tank reactors are carried out in the turbulent or transitional range.

The dissipation function (Bird et al. 2002) of a flow is the square of its shear rate. In the cylindrical coordinates, it can be determined on the basis of the fluid velocity derivatives from the equation:

5 $$\dot{\gamma }^{2} = 2\left[ {\left( {\frac{{\partial v_{r} }}{\partial r}} \right)^{2} + \left( {\frac{1}{r}\frac{{\partial v_{\theta } }}{\partial \theta } + \frac{{v_{r} }}{r}} \right)^{2} + \left( {\frac{{\partial v_{z} }}{\partial z}} \right)^{2} } \right] + \left[ {r\frac{\partial }{\partial r}\left( {\frac{{v_{\theta } }}{r}} \right) + \frac{1}{r}\frac{{\partial v_{r} }}{\partial \theta }} \right]^{2} + \left[ {\frac{1}{r}\frac{{\partial v_{z} }}{\partial \theta } + \frac{{\partial v_{\theta } }}{\partial z}} \right]^{2} + \left[ {\frac{{\partial v_{r} }}{\partial z} + \frac{{\partial v_{z} }}{\partial r}} \right]^{2} .$$

When the measurements of the fluid velocity are performed using a 2-D PIV technique, then the shear rate cannot be calculated directly from Eq. (5), since the third component of velocity is not measured. In order to estimate the value of the 3D shear rate some simplifying assumptions should be adopted. In this paper the rotational symmetry of the flow field was assumed, therefore all of the *θ* derivatives are zero. Furthermore, the assumption of constant angular velocity leads to a constant value of the $\frac{{v_{\theta } }}{r}$ and a zero value of the $\frac{{\partial v_{\theta } }}{\partial z}$. Applying the above assumptions in Eq. (5), a new equation is obtained for calculating approximate value of the shear rate as follows:

6 $$\dot{\gamma }_{2D} = \sqrt {2\left( {\frac{{\partial v_{r} }}{\partial r}} \right)^{2} +\; 2\left( {\frac{{v_{r} }}{r}} \right)^{2} + \;2\left( {\frac{{\partial v_{z} }}{\partial z}} \right)^{2} + \left( {\frac{{\partial v_{r} }}{\partial z} + \frac{{\partial v_{z} }}{\partial r}} \right)^{2} } .$$

Determination of $\dot{\gamma }$ distribution in the system considered is especially important during mechanical mixing of non-Newtonian fluid with elastic properties. For these fluids there is a limited value of the shear rate above which the elastic properties will start to dominate over the viscous properties. This value can be directly read from the rheological graph presenting the shear stress, *τ*, and the first difference of normal stress, *N*_1_, as a function of shear rate.

## Experimental

### Experimental rig

**Fig. 1:**
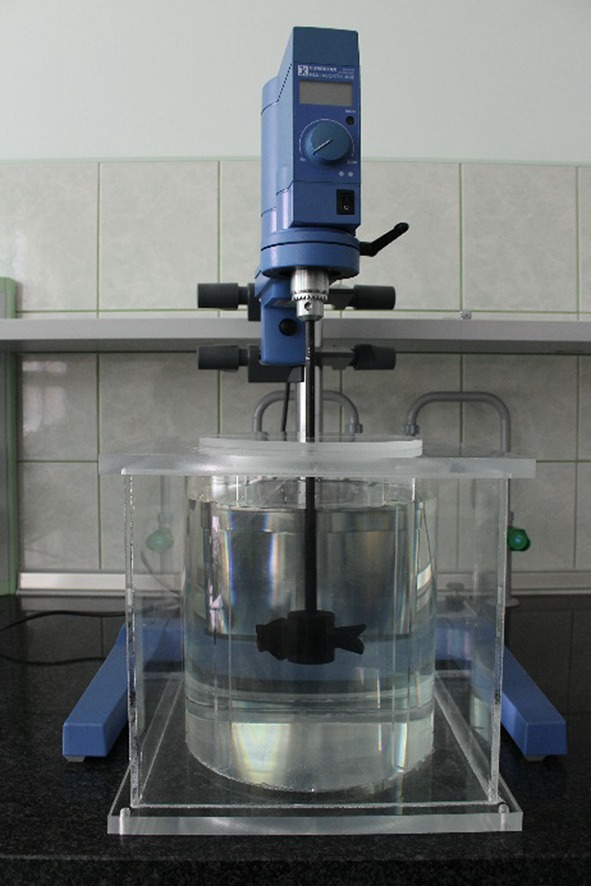
Experimental rig

The experimental study was performed in a flat-bottomed stirred tank (Fig. 1) with a diameter *T* = 0.222 m, equipped with four baffles (*B* = 0.1 *T*). Both the tank wall and baffles were made of Plexiglass to allow optical access from the sidewall. Thickness of the Plexiglass items was either 5 or 3 mm for the tank wall or baffles, respectively. The baffles were fixed in the cutters made in the tank bottom and the lid. The tank was filled with liquid (water or 0.2 wt% CMC) to a height *H* = *T*. A six-blade PMT-type impeller with a diameter *D* = 0.35 *T* was mounted centrally inside the tank. The impeller clearance was at a distance of *C* = 0.1 m from the tank bottom. The impeller was rotated at a constant frequency of *N* = 4.1 s^−1^ in the case of water, and either *N* = 4.1 or 8.2 s^−1^ in the case of CMC solution. Value of the mixing Reynolds number for water as the stirred fluid was *Re* = *ND* ^2^ *ρ*/*μ* = 2.43 × 10^4^, thus the flow was turbulent. For the non-Newtonian fluid, though, values of the mixing Reynolds number were estimated on the basis of the Metzner–Otto method, assuming the value of the Metzner–Otto coefficient equal to *k*_s_ = 11 (Jaworski and Nienow 1994). It led to obtained values of 499 or 1307 for the two tested impeller speeds. Thus the flow of the CMC solution was in the transient regime.

### CMC solution

Properties of the non-Newtonian fluids may differ depending on the type of solvent used as well as the type and concentration of the solute. In the case of aqueous solutions of carboxymethyl cellulose, which are often used as a model non-Newtonian fluids, it has been established (Jaworski 2010) that they may take the properties of Newtonian, shear-thinning, viscoelastic, or even thixotropic fluids, depending on the polymer concentration, the substitution degree and the cellulose chain length. A comprehensive rheological study (Jaworski 2010) relating to viscous and elastic properties of aqueous CMC solutions with different concentrations showed that in the tested range of concentrations the polymer solutions may have the normal stress, *N*_1_ (elastic properties), similar or higher than the shear stress, *τ* (viscous characteristics), even for low values of the shear rate. In addition, the author proposed an empirical correlation for calculating the normal stresses of the CMC solutions as a function of shear stress:

7 $$N_{1} = D\tau^{E} ,$$

where the *D* and *E* values were determined on the basis of the measurement results obtained for the CMC (Blanose 9H4) polymer solution with concentrations ranging from 0.1 to 0.5 wt% over a wide range of the shear rate. The coefficients are equal to *E* = 1.41 and *D* = 1.4 Pa^(1−*E*)^.

**Fig. 2:**
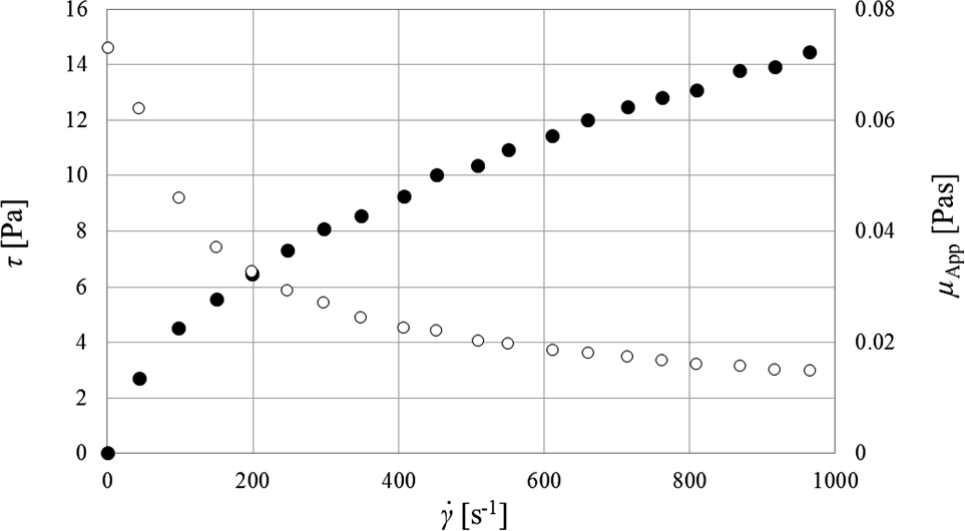
Shear stress (filled circle) and the apparent viscosity (unfilled circle) as a function of shear rate

An aqueous 0.2 wt% solution of the same CMC (Blanose 9H4 from Hercules) of high viscosity was used in this study as a model non-Newtonian fluid. Degree of substitution of this grade of CMC varies from 0.8 to 0.95 with its typical value approximately 0.9. The viscosity of 1% CMC 9H4 solution is in the range from 2500 to 4500 mPa s, while the weight average molecular weight of this solution is about *M*_w_ = 725,000 (Aqualon™ and Hercules 2006; Ashland 2016). Rheological measurements for the applied polymer solution were performed using a Rotovisco RT 10 rheometer (Haake) equipped with a coaxial cylinder sensor system. Inner diameter of the stator (*DG 41*) was equal to 43.4 mm and inner diameter of the rotor (*DG 41 DIN 53544*) was 36 mm. The temperature of the fluid was kept at the constant level of 20.5 ± 0.5 °C. Graphs of the measured shear stress, *τ*, and the apparent viscosity, *µ*_App_, as a function of shear rate, $\dot{\gamma }$, are shown in Fig. 2.

Based on the presented flow curve (Fig. 2), it can be concluded that the analyzed CMC solution is a shear-thinning fluid obeying the power law equation in the form $\tau = K \cdot \dot{\gamma }^{n}$, where *K* is the consistency coefficient and *n* is flow index. However, the power law does not include any elastic properties of the liquid, such as the first difference of normal stresses in the shear flow. However, the previous experimental study (Jaworski 2010) showed that 0.2 wt% CMC solution exhibits elastic properties. Considering that the shear stress characteristics of the CMC solution resulting from these experiments are similar to that from the literature (Jaworski 2010), it is assumed that the normal stress characteristic is also similar. Viscous properties of the CMC solution dominate over the elastic properties for the smallest values of the shear rate. However, with increasing shear rate the difference between the two stress types decreases, until finally the normal stress surpasses the shear stress. Moreover, the difference between *N*_1_ and *τ* would still increase with increasing $\dot{\gamma }$. Therefore, it was decided to take the elastic properties of the liquid into account during the flow analysis of the CMC solution in a stirred tank.

Based on the literature (Jaworski 2010) and correlation (7), for the tested CMC solution, a value of 0.44 Pa of the first difference of normal stress is obtained as equal to the shear stress. Above this threshold value the normal stress surpasses the shear stress. Using the flow characteristics of the CMC solution (Fig. 2), the threshold shear rate of $\dot{\gamma }_{\text{c}} \cong 8$ s^−1^ was calculated above which the normal stress *N*_1_ exceeds the shear stress *τ*.

### PIV setup

For the velocity field measurements, the 2-D PIV technique was applied. PIV provides information about instantaneous velocity field in a selected plane by imaging the scattering of laser light from the tracer particles added into the fluid. The main components of the PIV system are: laser, CCD camera, synchronizer and computer with software. During the measurements the laser light in the form of the plane illuminates the flow field. The laser light illuminates the tracer particles which follow the flow, scattering light. Next, the CCD camera captures images of the plane illuminated by the laser sheet and records movements of the seeding particles. The images captured in the known interval of time are then sent to the computer, where they are analyzed. More detailed information about correlation of the images recorded during PIV measurements can be found in the literature (Keane and Adrian 1990, 1991; Prasad 2000; Mavros 2001; Raffel et al. 2007). The use of laser light in the form of plane enables collection of the data in a multi-point mode, which practically gives instantaneous information about the generated velocity field in the field of view of the tested system. Consequently, a preliminary evaluation of the results can be done even during the measurements.

**Fig. 3:**
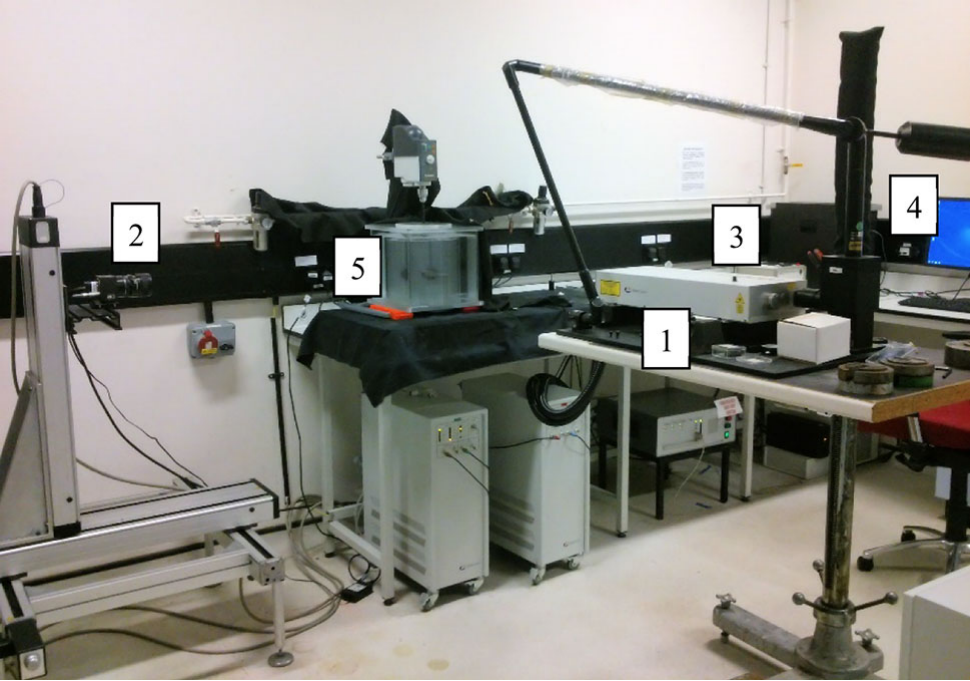
PIV system: 1—laser, 2—CCD camera, 3—synchronizer, 4—computer, 5—stirred tank

In this study a 2-D PIV system was used (Fig. 3), which consists of the Litron Lasers Nano L 50–100 PIV Pulsed Nd:YAG laser with a wavelength of 532 nm and the TSI PowerView™ Plus CCD camera (4 MPx). The camera was mounted on a computer-controlled traverse. The laser and camera were controlled by the synchronizer TSI Laserpulse 610030. As tracer particles, silver-coated hollow glass spheres with an average diameter of 10 μm from Dantec Ltd, UK, were used. The time period between images capture was set up to 400 ms. In the case of water 100 instantaneous values of local velocity were collected, while in the case of 0.2% CMC the sample number was equal to 60. Image analysis was performed using the TSI INSIGHT™ 4G software and FFT cross-correlation. The images were processed using Recursive Nyquist Grid. Descending area of displacement search was applied, with final interrogation area of 32 × 32 pixels and 25% overlap. Due to the symmetry of the mixing tank the vertical imaging plane chosen was aligned through the impeller axis, illuminating one side of the vessel from the wall to the impeller shaft. The measurement plane was located at an angle of 45° between neighboring baffles or in the line of baffles. One image frame covered the entire analyzed plane from the tank bottom up to the free surface of the liquid.

## Results and discussion

### Mean velocity

**Fig. 4:**
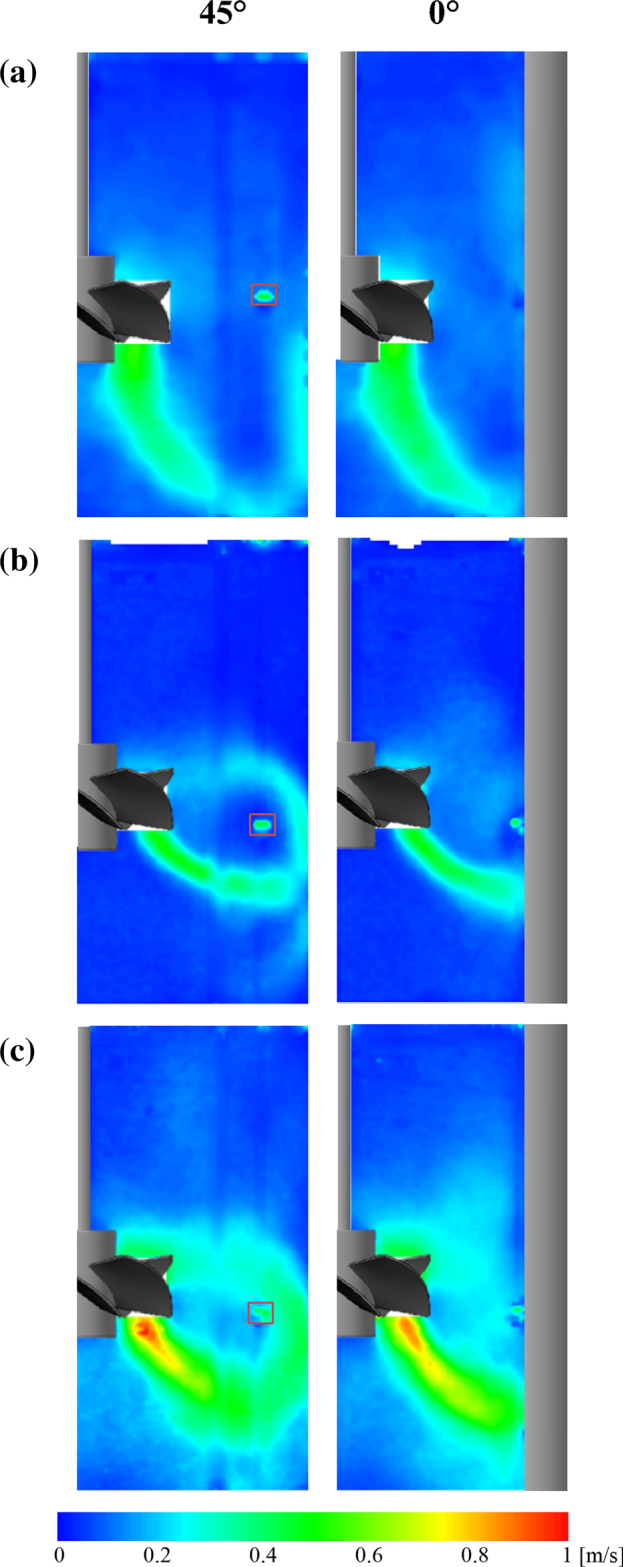
Maps of the mean velocity: **a** water, **b** 0.2% CMC, *N* = 4.1 s^−1^, **c** 0.2% CMC, *N* = 8.2 s^−1^

**Fig. 5:**
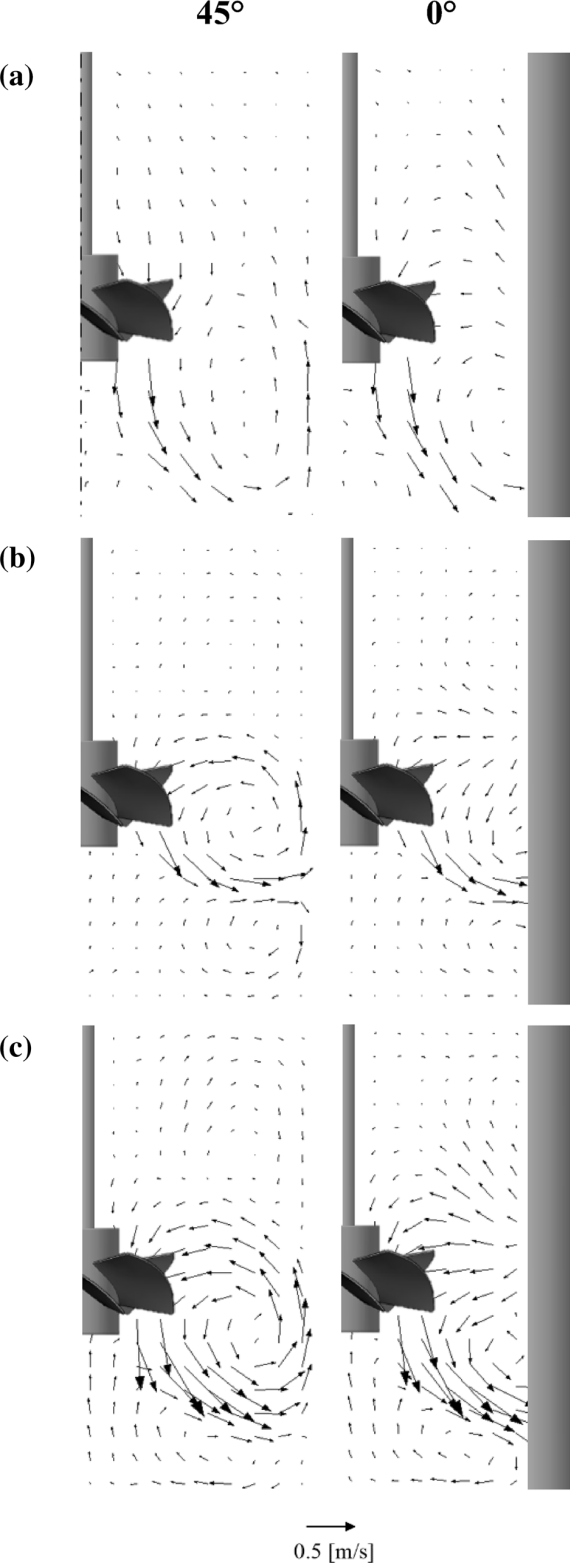
Vectors of the mean velocity: **a** water, **b** 0.2% CMC, *N* = 4.1 s^−1^, **c** 0.2% CMC, *N* = 8.2 s^−1^

On the basis of PIV measurements the local instantaneous values of the mean fluid velocity were obtained and ensemble averaged for different angular positions of the impeller blades against the measurement plane. The averaged values of the mean fluid velocity are presented graphically as maps (Fig. 4) and vectors (Fig. 5) for a mid-plane located in two angular positions against the baffles: 45° and 0°. In the presented maps a small area was found (marked with a frame) which could give wrong information about generated velocity field, and consequently about shear rate field. This area was not taken into consideration during analysis.

Analyzing the velocity maps for the Newtonian fluid (water, *N* = 4.1 s^−1^), typical shown in Fig. 4a, it was found that the mean velocities have values in the range from 0 to 0.52 m s^−1^, reaching a maximum value just below the impeller blades. The maximum value of the water mean velocity was equal to 0.52 *v*_TIP_, with the impeller peripheral speed defined as *v*_TIP_ = *πDN*. This value closely matches that from the literature LDA data (Jaworski and Nienow 1994). In the case where the process medium was the non-Newtonian 0.2 wt% CMC solution, the fluid mean velocity (Fig. 4b, c) had values in the range from 0 to 0.47 m s^−1^ for *N* = 4.1 s^−1^, while a twofold increase of the maximum impeller speed (to *N* = 8.2 s^−1^) caused less than twofold increase of the fluid velocity (0 to 0.87 m s^−1^). The maximum values of the non-Newtonian fluid mean velocity were 0.47 and 0.44 *v*_TIP_ for the *N* = 4.1 and 8.2 s^−1^, respectively. Analogous values calculated from the LDA measurements (Jaworski and Nienow 1994) for the same impeller speeds were, respectively, 0.43 and 0.45 *v*_TIP_. Thus it could be safely assumed that the maximum values of the measured standardized velocity of the CMC solution equal approximately 0.44, significantly lower than those for water.

In order to quantitatively compare the results obtained from the PIV with the LDA literature data, a mean square deviation, *δ*, between standardized values of the two velocity components was calculated from Eq. (8):

8 $$\delta = \sqrt {\frac{{\mathop \sum \nolimits (V_{{{\text{I,LDA}},j}} - V_{{{\text{I,PIV}},j}} )^{2} }}{(n - 1)}} \times 100\% \quad {\text{for}}\quad j = 1 \ldots \,n,$$

9 $${\text{where}}:\quad V_{\text{I}} = \frac{{\bar{v}_{i} }}{\pi DN}\quad {\text{for}}\quad i = z, r.$$

The results are summarized in Table 1. The maximum deviation value between the two standardized mean velocity components for water did not exceed 4.9 and 6.7% for the CMC. This gives good agreement between the results obtained with both experimental techniques.

**Table 1:** Percentage value of the mean square deviation of two components of the mean fluid velocity between LDA measurements (Jaworski and Nienow 1994) and present PIV measurements

Fluid (*N*, s^−1^)	*V*_Z_	*V*_R_
Water (4.1)	4.2	4.9
0.2% CMC (8.2)	3.3	6.7

**Fig. 6:**
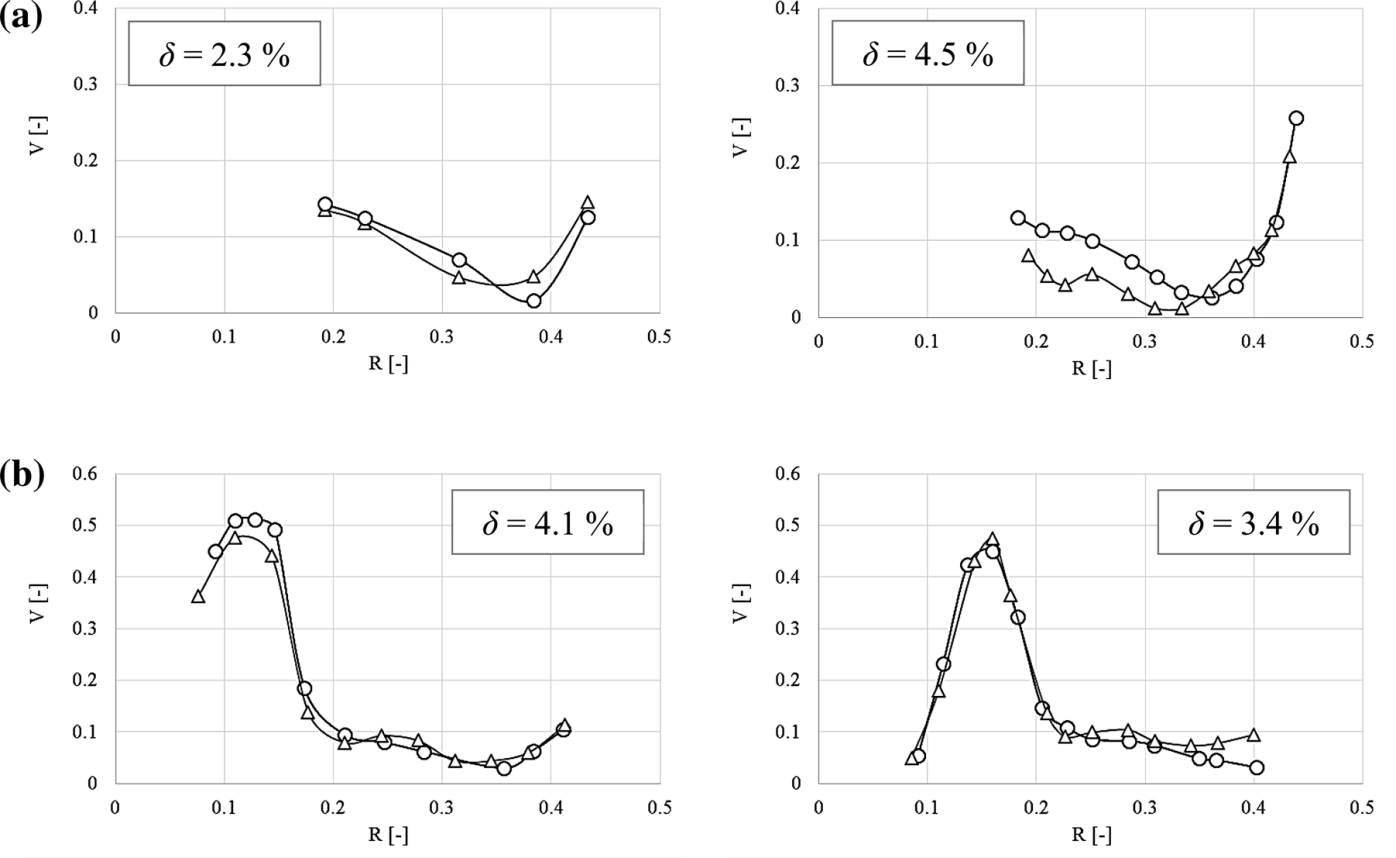
Profiles of the standardized mean velocity at the level: **a** *z* = 0 mm, **b** *z* = − 17 mm [PIV (circle), LDA (triangle), LDA data (Jaworski et al. 1996)]

Additionally, profiles of the standardized mean velocity along dimensionless radius (*R* = *r*/*T*) for water and 0.2 wt% CMC (*N* = 8.2 s^−1^), obtained from the two measurements techniques were plotted. Sample of such profiles, at level *z* = 0 mm and *z* = −17 mm, where *z* = 0 indicates the impeller center, as well as values of mean square deviation of the mean fluid velocity between LDA and PIV data (Eq. 8) are presented in Fig. 6. Analyzing the presented profiles it was found that results from PIV are in good accordance with those from LDA.

Distribution of the velocity vectors (Fig. 5) is also consistent with the literature LDA data (Jaworski and Nienow 1994) obtained for the analogous system, confirming the qualitative similarity of the LDA and PIV results.

Analyzing the velocity vectors for water (Fig. 5a) it was found that the primary circulation loop, which encompasses the rotating impeller spreads from the center of the impeller to the tank bottom. There were also two secondary circulation loops, induced by motion of the fluid in a radial–axial plane. One of them was formed underneath the impeller hub, the second in the top of the tank at the free surface of the water. The flow intensity of the secondary circulation loops was much lower than that in the primary loop. It can be concluded, that in the studied system and for the specified process parameters, the PMT impeller gives a very good axial circulation. The circulation flow number, *K*_C_, defined as:

10 $$K_{\text{C}} = \frac{{Q_{\text{C}} }}{{ND^{3} }} \quad{\text{for }} \quad Q_{\text{C}} = { \hbox{max} }\left\{ {Q_{z/r} } \right\},$$

11 $${\text{where}}\quad Q_{z} \left( {z_{i} } \right) = 2\pi \mathop \smallint \limits_{{r_{1} }}^{{r_{2} }} r\left( {\bar{v}_{z} } \right)_{{z_{i} }} {\text{d}}r \quad Q_{r} \left( {r_{i} } \right) = 2\pi r_{i} \mathop \smallint \limits_{{z_{1} }}^{{z_{2} }} \left( {\bar{v}_{r} } \right)_{{r_{i} }} {\text{d}}z,$$

was equal to *K*_C,PIV,water_ = 1.50 and is similar to the literature for the analogous system where *K*_C,LDA,water_ = 1.55 (Jaworski and Nienow 1994).

In the case of CMC solution, with increasing the impeller speed the primary circulation loop size also increased. Compared to the water case, the loop was located closer to the impeller and spread in the distance about 50 or 70 mm below the center of the impeller, for the impeller speed of 4.1 or 8.2 s^−1^, respectively. Two secondary circulation loops were here much bigger than for water. Distribution of the velocity vectors for the non-Newtonian fluid (Fig. 5b, c) suggests that the PMT-type impeller pumped the fluid axially downwards, but mainly in the radial direction to the tank wall. It can be concluded that for the tested range of the process parameters the axial–radial circulation generated by the PMT-type impeller in the non-Newtonian fluid was less than that for the Newtonian fluid. This is confirmed by the circulation flow number, which has a less value than that for water and is equal to *K*_C,PIV,CMC_ = 0.96 or 1.12 for *N* = 4.1 or 8.2 s^−1^, respectively. Value of the flow number calculated from the LDA data for the CMC solution is available in the literature (Jaworski et al. 1996) and for *N* = 8.2 s^−1^ is equal to *K*_C,LDA,CMC_ = 1.20.

### Shear rate of the CMC solution

**Fig. 7:**
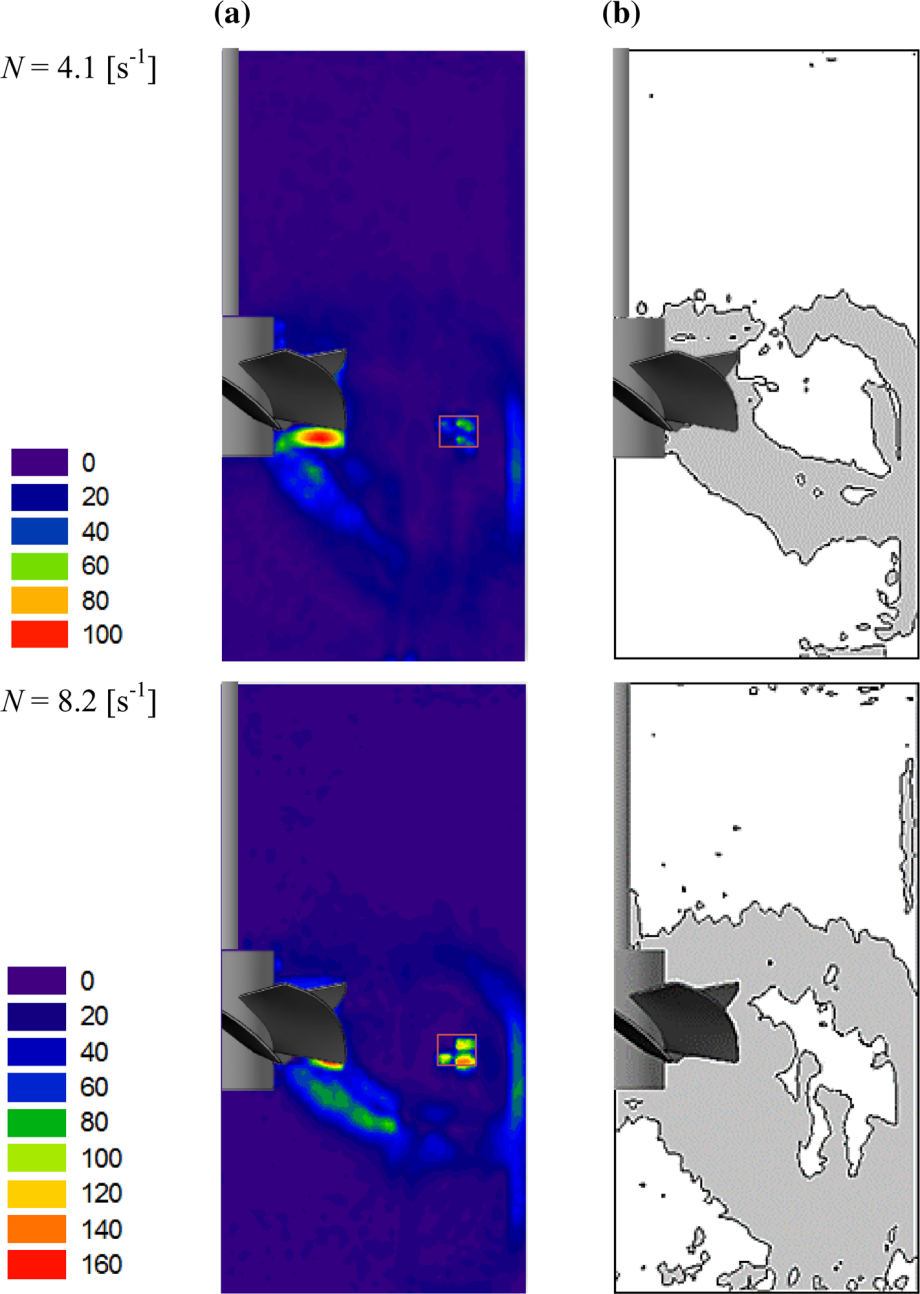
Shear rate of the CMC solution for two impeller speeds: **a** maps, **b** isolines of $\dot{\gamma }_{2D,c} = 8\,{\text{s}}^{ - 1}$

The presented set of PIV measurements was also used to assess the importance of elastic forces in the stirred liquid. Based on the calculated values of the fluid velocity derivatives: $\frac{{\partial v_{r} }}{\partial r}$, $\frac{{\partial v_{z} }}{\partial z}$, $\frac{{\partial v_{r} }}{\partial z}$, $\frac{{\partial v_{z} }}{\partial r}$ and $\frac{{v_{r} }}{r}$, approximate local values of the 2D shear rate were calculated from Eq. (6). Then, the distributions of the shear rate, $\dot{\gamma }_{{2{\text{D}}}}$, in a mid-plane between two adjacent baffles were constructed and are shown in Fig. 7a. Isolines of the constant value $\dot{\gamma }_{{2{\text{D,c}}}} = 8$ s^−1^, equal to the threshold, were also presented (Fig. 7b). The shaded area between the isolines indicates the region in which the normal stress surpasses the shear stress.

The obtained maps of $\dot{\gamma }_{{2{\text{D,c}}}}$ (Fig. 7a) are qualitatively similar to the maps of mean velocity (Fig. 4) for the corresponding impeller speeds. Analyzing the results it was found that the maximum values of the shear rate were observed just below the impeller blades and were equal to 99.9 or 163.6 s^−1^ for the *N* = 4.1 or *N* = 8.2 s^−1^, respectively. When water was used as a process liquid then approximately twofold lower value of the shear rate ($\dot{\gamma }_{{2{\text{D,max}}}}$ = 49.5 s^−1^) was obtained for the impeller speed *N* = 4.1 s^−1^. The increased values of the shear rate in CMC solution were also observed near the tank wall; however, they were not as high as those in the impeller area. When taking into account isolines of the $\dot{\gamma }_{2D,c} = 8$ s^−1^ (Fig. 7b), it should be noted that elastic properties dominate over the viscous in a large part of the stirred viscoelastic solution. Thus, for *N* = 4.1 s^−1^ the shaded area occupies about 20% of the presented plane, while for *N* = 8.2 s^−1^ it grows up to 39%.

It is very important to note that due to the 2-D PIV used, values of the shear rate, $\dot{\gamma }$, were approximately determined as $\dot{\gamma }_{{2{\text{D}}}}$, on the basis of only 5 of 9 components of the velocity gradient—for two velocity components and two directions. In fact, $\dot{\gamma }$ should be calculated from Eq. (5) for all three components of the liquid velocity and for three directions. Because all of the components of this equation are in the power of 2, it means that the values estimated from Eq. (6) $\dot{\gamma }_{{2{\text{D}}}}$ are underestimated. Consequently, it can be concluded that the real areas where the normal force will exceed the shear force will be larger than those presented in Fig. 7b.

## Conclusions

The velocity field measurements for the Newtonian (water) and non-Newtonian (0.2 wt% CMC) fluids in a stirred tank equipped with a high-speed PMT-type impeller were presented. The experimental study was performed using a 2-D PIV technique and positively compared to the literature velocity data from LDA.

Based on the experimental study results, it was found that, both the rheological properties of the stirred medium and an increase of the impeller speed, affect the generated velocity field as well as the size and location of the circulation loops. The radial–axial mean fluid velocity reached maximum values just below the impeller blades and they were equal to $\bar{v}_{ \hbox{max} }$ = 0.52, 0.51, 0.87 m/s, respectively, for water, CMC solution and *N* = 4.1 s^−1^ and CMC solution and *N* = 8.2 s^−1^. The applied impeller produced a larger primary circulation loop for the Newtonian fluid than for the non-Newtonian solution.

During the processing of the results and their analysis, emphasis was placed on generation of the shear rate distribution for the non-Newtonian fluid in order to indicate area where the normal stress surpasses the shear stress. It was estimated that within the analyzed plane, located at an angle of 45° between adjacent baffles, this area occupied 20 and 39% for the *N* = 4.1 and *N* = 8.2 s^−1^, respectively. It was also highlighted that the size of the indicated area may be underestimated due to the applied measurement technique (2-D PIV). Nevertheless, based on the performed study it was concluded that the elastic properties can dominate over the viscous in a considerable volume of the fluid that was characterized by intensive circulation. Therefore, it is concluded that elastic forces should be taken into account during numerical qualitative and quantitative flow analyses of such mildly viscoelastic fluids.

## List of symbols

*B*: Baffle width, m
*D*: Impeller diameter, m
*D, E*: Empirical constants in Eq. (7)
*k*_s_: Metzner–Otto coefficient
*K*: Consistency index, Pa s^*n*^
*K*_C_: Circulation flow number
*N*: Power-law index
*N*: Impeller speed, s^−1^
*N*_1_: First normal stress difference, Pa
*Q*_C_: Volumetric flow rate, m^3^ s^−1^
*R*: Radial coordinate, m
*R*: Dimensionless radial coordinate
*Re*: Reynolds number
*T*: Tank diameter, m
$v$: Instantaneous velocity, ms^−1^
$v'$: Fluctuating velocity, ms^−1^
$\bar{v}$: Mean velocity, ms^−1^
$v_{\text{TIP}}$: Impeller peripheral speed, ms^−1^
$V$: Dimensionless mean velocity
*We*: Weissenberg number

## Greek letters

$$\dot{\gamma }$$: Shear rate, s^−1^
*δ*: Mean square deviation, %
*μ*: Dynamic viscosity of agitated liquid, Pa s
*μ*_App_: Apparent viscosity of non-Newtonian liquid, Pa s
*ρ*: Density of agitated liquid, kg m^−3^
*τ*: Shear stress, Pa
*σ*: Normal stress, Pa

## Subscripts

*C*: Indicates the threshold value
*R*: Radial coordinate
*Z*: Axial coordinate
*θ*: Tangential coordinate
max: Indicates the maximum value

## Abbreviations

CMC: Carboxymethyl cellulose
LDA: Laser Doppler anemometry
PIV: Particle image velocimetry
